# Effects of Virtual Reality–Based Intervention Combined With Conventional Therapy on Upper-Limb Function in Children With Hemiplegic Cerebral Palsy: Systematic Review and Meta-Analysis of Randomized Controlled Trials

**DOI:** 10.2196/98694

**Published:** 2026-08-26

**Authors:** Chengqian Feng, Yang Li, Letian Cheng, Baizhen Xiong, Zhicheng Lin, Caihua Huang

**Affiliations:** 1 Department of Physical Education Xiamen University Xiamen, Fujian China; 2 School of Clinical Medicine Zhongshan College of Dalian Medical University Dalian, Liaoning China; 3 Department of Physical Education Xiamen University of Technology Xiamen, Fujian China

**Keywords:** virtual reality, hemiplegic cerebral palsy, upper limb, digital health, rehabilitation

## Abstract

**Background:**

Hemiplegic cerebral palsy (CP) commonly affects upper-limb function in children and may limit functional use of the affected arm and hand. Virtual reality (VR)–based rehabilitation can provide interactive, feedback-based, and task-oriented practice, but whether adding VR-based intervention to conventional therapy provides additional benefits remains uncertain. Previous reviews have often included broader CP populations, mixed comparators, or broad upper-limb outcome labels, limiting domain-specific interpretation.

**Objective:**

This systematic review and meta-analysis evaluated VR-based intervention combined with conventional therapy versus conventional therapy alone for upper-limb–related outcomes in children with hemiplegic CP.

**Methods:**

This review was prospectively registered with PROSPERO (CRD420261486172). PubMed, Embase, Web of Science, and the Cochrane Central Register of Controlled Trials were searched from inception to April 15, 2026. Eligible randomized controlled trials included children aged ≤12 years with hemiplegic CP and compared VR-based or interactive game–based intervention plus conventional therapy with conventional therapy alone. Outcomes were grouped into upper-limb activity, dexterity, motor control and movement quality, activities of daily living (ADLs), and grip strength. Risk of bias was assessed using the revised Cochrane risk-of-bias 2 tool. Random-effects meta-analyses with Hartung-Knapp-Sidik-Jonkman adjustment were performed; Nagashima-corrected prediction intervals (PIs) were calculated where applicable; and certainty of evidence was assessed using Grading of Recommendations Assessment, Development, and Evaluation (GRADE).

**Results:**

Thirteen randomized controlled trials involving 458 children were included. Pooled estimates generally favored VR combined with conventional therapy, but no outcome reached statistical significance; effect estimates were as follows: upper-limb activity (standardized mean difference [SMD] 0.28, 95% CI −0.07 to 0.64), dexterity (SMD 0.26, 95% CI −0.34 to 0.86), motor control and movement quality (SMD 0.29, 95% CI −0.22 to 0.80), ADLs (SMD 0.17, 95% CI −0.22 to 0.55), and grip strength (mean difference 0.89, 95% CI −1.93 to 3.72). PIs also crossed the null value for all outcomes, ranging from −0.51 to 1.04 for upper-limb activity and from −4.89 to 6.45 for grip strength, suggesting variable effects across settings. Heterogeneity was low to moderate for most outcomes but substantial for grip strength. Most trials had some risk-of-bias concerns. Certainty of evidence was low for all outcomes except grip strength, which was very low.

**Conclusions:**

VR-based intervention combined with conventional therapy did not provide clear evidence of additional benefits over conventional therapy alone. Although effects generally favored the combined intervention, CIs, PIs, risk-of-bias concerns, and low to very low certainty of evidence support cautious interpretation. VR may be considered a potentially engaging adjunct, but current evidence does not support its use as a replacement for therapist-led rehabilitation or as a definitively effective intervention. Larger, adequately powered trials with clearer reporting of VR characteristics, intervention dose, task specificity, and longer follow-up are needed.

**Trial Registration:**

PROSPERO CRD420261486172; https://www.crd.york.ac.uk/PROSPERO/view/CRD420261486172

## Introduction

### Background

Conventional therapies, including constraint-induced movement therapy and neurodevelopmental therapy, are widely applied in neurological rehabilitation [1]. Although clinically effective, traditional interventions are often associated with high repetitiveness and low engagement, frequently leading to poor participation among pediatric patients. This may reduce long-term adherence in the pediatric population, thereby compromising the effectiveness of clinical translation [2]. In recent years, virtual reality (VR)–based rehabilitation has been increasingly applied in children with cerebral palsy (CP) as a potential adjunct to conventional therapy. By providing interactive, game-like tasks with real-time visual, auditory, or sensorimotor feedback, VR systems may enhance motivation, support repetitive task-oriented practice, and facilitate motor learning [3-6].

Current systematic reviews have examined VR-based rehabilitation in children with CP, but their scopes, populations, intervention characteristics, comparator conditions, and outcome measures vary substantially. Some reviews have focused on upper-limb outcomes but included broader CP populations and age ranges. For example, Burin-Chu et al [7] included children and young adults with unilateral or bilateral CP and found that VR combined with conventional therapy improved upper-limb activity compared with conventional therapy alone, although the certainty of evidence was low to very low. Alrashidi et al [8] also focused on upper-limb motor function in children with CP but reported inconsistent findings across randomized trials, suggesting that the effect of VR on upper-limb rehabilitation remains uncertain. Other reviews have examined broader functional outcomes, such as balance, gross motor function, and activities of daily living (ADLs), rather than domain-specific upper-limb outcomes [9-11]. More recently, Ibrahim et al [12] specifically examined children with hemiplegic CP and included upper-limb–related outcomes; however, the intervention and comparator conditions were mixed, including comparisons of VR with no intervention, conventional therapy, or alternative rehabilitation approaches. At the same time, previous reviews often used broad outcome labels such as upper-limb function or hand function, without clearly distinguishing the different constructs captured by individual outcome measures. Therefore, the adjunctive effect of VR combined with conventional therapy compared with conventional therapy alone on specific upper-limb functional domains in children with hemiplegic CP remains unclear.

### Rationale

CP is one of the most prevalent nonprogressive central nervous system disorders in children. It is characterized by impairments of movement and posture and may be accompanied by cognitive, sensory, and perceptual deficits [3]. As the leading cause of motor disability in children, CP has an estimated prevalence of 3.3 per 1000 children in the United States [13]. Among all CP subtypes, hemiplegic CP is one of the most common clinical presentations in children with CP [14]. Hemiplegic CP is characterized by a motor lesion predominantly affecting one side of the body, typically involving the arm [15]. The central nervous system lesions underlying hemiplegic CP are predominantly confined to one cerebral hemisphere. While such unilateral brain injury may stem from various etiologies, a common cause is anterior cerebral artery infarction occurring in the third trimester of pregnancy [16]. Nearly half of children with hemiplegic CP present greater impairment in the upper extremities, especially in hand function, than in the lower extremities [15,17]. In children with hemiplegic CP, upper-limb involvement commonly includes impaired hand use, reduced selective control, abnormal grasp patterns, and limited wrist or finger movement [18]. Because of unilateral weakness and impaired bimanual coordination, children may rely more heavily on their less affected limb during daily tasks, potentially contributing to reduced spontaneous use of the affected hand or learned nonuse [19]. These impairments can limit object manipulation, bimanual activities, self-care, play, and school participation [20]. Therefore, interventions targeting upper-limb use, coordination, and functional task practice are clinically important in this population.

### Objectives

To address this research gap, this systematic review and meta-analysis synthesized and critically appraised evidence from randomized controlled trials to examine the adjunctive role of VR-based intervention combined with conventional therapy in upper-limb rehabilitation for children with hemiplegic CP. This review restricted the population to children with hemiplegic CP; limited the comparison to VR combined with conventional therapy versus conventional therapy alone; and classified upper-limb–related outcomes more precisely based on the International Classification of Functioning, Disability and Health (ICF) framework and instrument-specific constructs. This focused approach allows a clearer assessment of whether adding VR-based intervention to conventional therapy improves upper-limb recovery in this population.

## Methods

### Overview

The protocol for this study was prospectively registered with PROSPERO (CRD420261486172). The review methods were guided by the Cochrane Handbook for Systematic Reviews of Interventions. Two minor deviations from the registered protocol occurred: the search strategy was expanded to improve retrieval sensitivity, and the statistical model was revised to random-effects meta-analysis with Hartung-Knapp-Sidik-Jonkman (HKSJ) adjustment to better account for expected clinical and methodological heterogeneity.

### Eligibility Criteria

Eligible participants were required to have a clinical diagnosis of hemiplegic CP and were limited to children aged ≤12 years. This prespecified age threshold was adopted to target a specific pediatric cohort and reduce heterogeneity associated with developmental stage. Studies were excluded if participants had severe visual impairment or significant cognitive deficits hindering effective engagement in VR-based rehabilitation, which relies on visual feedback, visuomotor integration, task understanding, and active involvement. For studies enrolling participants with different levels of visual or cognitive dysfunction, inclusion was determined by their ability to follow instructions and complete VR interventions independently. Mild and moderate impairments were not set as exclusion criteria unless they impeded participation. Studies involving individuals with acquired neurological disorders such as traumatic brain injury were also excluded.

Eligible interventions included VR-based interventions delivered as an adjunct to conventional therapy. VR-based interventions were defined as interactive virtual or screen-based systems that provide real-time visual, auditory, or sensorimotor feedback and require active upper-limb movement and interaction between the participant and the system during therapeutic tasks [21]. According to immersion level, interventions were classified as immersive VR, semi-immersive VR, or nonimmersive VR [22]. Conventional therapy referred to standard rehabilitation approaches used in the original trials, such as physical therapy, occupational therapy, neurodevelopmental treatment, task-oriented training, stretching, strengthening exercises, or routine motor rehabilitation [1,23,24]. The comparator was conventional therapy alone. Studies were excluded if VR was used as a stand-alone intervention, if the control group did not receive conventional therapy alone, or if the intervention involved only passive video viewing without interactive components.

Eligible studies were required to report sufficient numerical data for at least 1 relevant upper-limb motor or functional outcome. For quantitative synthesis, outcomes were grouped into 5 predefined domains: upper-limb activity, dexterity, motor control and movement quality, ADLs, and grip strength; detailed operational definitions and measurement instruments for each domain are described in the Data Items section. Studies were excluded if they did not report relevant standardized motor or functional outcomes; focused only on feasibility, usability, or immersion experience; or provided insufficient extractable or calculable data. Only randomized controlled trials were included. Reviews, meta-analyses, case reports, editorials, nonrandomized studies, conference abstracts without accessible full-text data, and non-English publications were excluded.

### Information Sources

A comprehensive literature search was conducted in MEDLINE via PubMed, Embase via Elsevier, Web of Science Core Collection via Clarivate Web of Science, and the Cochrane Central Register of Controlled Trials via the Cochrane Library. No restriction on publication date was applied. In addition, the reference lists of included studies and relevant reviews were manually screened to identify additional eligible studies. No study registries were searched.

### Search Strategy

The literature search process was reported in strict accordance with the PRISMA-S (Preferred Reporting Items for Systematic Reviews and Meta-Analyses literature search extension) guidelines [25]. Search strategies were developed separately for each database using database-specific controlled vocabulary terms and free-text terms. The search combined terms related to the population, intervention, outcomes, pediatric population, and study design, including terms for hemiplegic CP, unilateral CP, VR, exergaming, interactive video games, Nintendo Wii, Xbox Kinect, upper-limb function, hand function, children, and randomized or controlled trials. Controlled vocabulary terms were used where available, including MeSH terms in MEDLINE or PubMed and the Cochrane Library, and Emtree terms in Embase. All databases were searched from inception to April 15, 2026. The search was restricted to articles published in English. No formal peer review of the search strategy was conducted using the PRESS (Peer Review of Electronic Search Strategies) checklist or by an information specialist. The detailed search string and specific keywords used for each database are provided in Multimedia Appendix 1.

### Selection Process

Initially, all retrieved records were imported into reference management software EndNote (version 2025.3.1; Clarivate) to identify and remove duplicates. Subsequently, 2 independent reviewers conducted a blinded, duplicate screening of titles and abstracts to exclude studies that clearly did not meet the predefined inclusion criteria. In the second stage, the full-text versions of the remaining articles were thoroughly retrieved and scrutinized to confirm their final eligibility. Any discrepancies between the 2 reviewers during the selection process were resolved through discussion and consensus; if a disagreement persisted, a third senior researcher was consulted to reach a final decision. No automation tools, machine learning classifiers, crowdsourcing methods, or previously screened datasets were used in the study selection process.

### Data Collection Process

Data were extracted independently by 2 reviewers using a standardized data extraction form. Extracted information was cross-checked between reviewers, and any discrepancies were resolved through discussion. Study authors were not contacted for missing, incomplete, or unclear data. When the necessary numerical data could not be extracted directly from the published report or calculated from the available information, the corresponding outcome was excluded from the quantitative synthesis. No automation tools were used in the data collection process.

### Data Items

The predefined outcome domains were upper-limb activity, dexterity, motor control and movement quality, ADLs, and grip strength. Upper-limb activity referred to the actual use or participation of the affected upper limb in functional or bimanual tasks; dexterity referred to the speed, precision, and efficiency of hand manipulation; motor control and movement quality referred to selective initiation, coordination, and regulation of upper-limb movements and the qualitative performance of the affected limb during functional use; ADLs referred to functional independence in everyday tasks; and grip strength referred to maximal voluntary handgrip force. Outcome instruments were assigned to each domain according to their primary measurement construct. The detailed classification of outcome instruments is provided in Multimedia Appendix 2.

For each outcome, postintervention means, SDs, and sample sizes were extracted for the intervention and control groups. Other extracted variables included first author, publication year, country, study design, CP type, age, sex, Manual Ability Classification System (MACS) or Gross Motor Functional Classification System (GMFCS) classification, sample size, outcome instruments, intervention and comparator conditions, VR device, virtual scenario, VR immersion type, session duration, frequency, intervention period, treatment setting, supervision status, and adverse events.

### Study Risk-of-Bias Assessment

The methodological quality of the included randomized controlled trials was independently evaluated by 2 reviewers using the revised Cochrane risk-of-bias 2 tool (RoB 2). Potential bias was assessed across five domains: (1) randomization process, (2) deviations from intended interventions, (3) missing outcome data, (4) measurement of the outcome, and (5) selection of the reported result. Each domain was rated as “low risk,” “some concerns,” or “high risk.” Any disagreements were resolved through consensus or by a third senior researcher [26].

### Effect Measures

For continuous outcomes measured using different instruments or scales, standardized mean differences (SMDs) corrected for small-sample bias, expressed as Hedges *g*, were used. When outcomes were reported using the same measurement unit across studies, mean differences (MDs) were used to improve clinical interpretability [27].

### Synthesis Methods

Outcome measures were grouped into the predefined domains described in the Eligibility Criteria and Data Items sections. For outcomes in which lower scores indicated better performance, data were directionally transformed so that positive effect estimates consistently favored VR-based intervention combined with conventional therapy. Forest plots were used to display individual study effects and pooled estimates, while study characteristics, intervention details, and risk-of-bias judgments were summarized in tables and figures. No double counting occurred in the meta-analyses [28].

Random-effects models were used for all analyses because clinical and methodological heterogeneity was expected across trials [29-33]. The HKSJ adjustment was applied to estimate CIs and *P* values for pooled effects [34]. Statistical heterogeneity was assessed using the *I*² statistic, Cochran Q test, and the between-study variance τ². The *I*² statistic was used to describe the proportion of observed variability attributable to between-study heterogeneity, but it was not interpreted as an absolute measure of the dispersion of true effects across populations or settings. Where a sufficient number of studies was available, 95% prediction intervals (PIs) were calculated to describe the expected range of true effects in future comparable studies. PIs were interpreted cautiously when fewer than 10 studies were included and were not emphasized when fewer than 5 studies were available, as estimates of between-study dispersion are unstable with a small number of studies [35]. When PIs were calculated, the Nagashima-Noma-Furukawa bootstrap approach was used to obtain Nagashima-corrected 95% PIs, which account for uncertainty in the estimation of between-study variance and are more appropriate when the number of studies is small [36]. Subgroup analyses and metaregression were not performed because the number of studies per outcome was limited. Sensitivity analyses using the leave-one-out method were performed for all outcomes to ensure the stability of the findings [29].

### Reporting Bias Assessment

Funnel plots and Egger or Begg tests were used to assess publication bias and small-study effects only when at least 10 studies were available for a given outcome. These methods were not applied when fewer than 10 studies were available because tests for funnel plot asymmetry have low power with a small number of studies [37].

### Certainty Assessment

The certainty of evidence for each main outcome was assessed using the Grading of Recommendations Assessment, Development, and Evaluation (GRADE) approach [38]. Evidence from randomized controlled trials was initially rated as high certainty and was downgraded when concerns were identified in 5 domains: risk of bias, inconsistency, indirectness, imprecision, and publication bias. The certainty of evidence was categorized as high, moderate, low, or very low. GRADE assessments were conducted independently by 2 reviewers, and disagreements were resolved through discussion or consultation with a third reviewer.

## Results

### Study Selection

**Figure 1:**
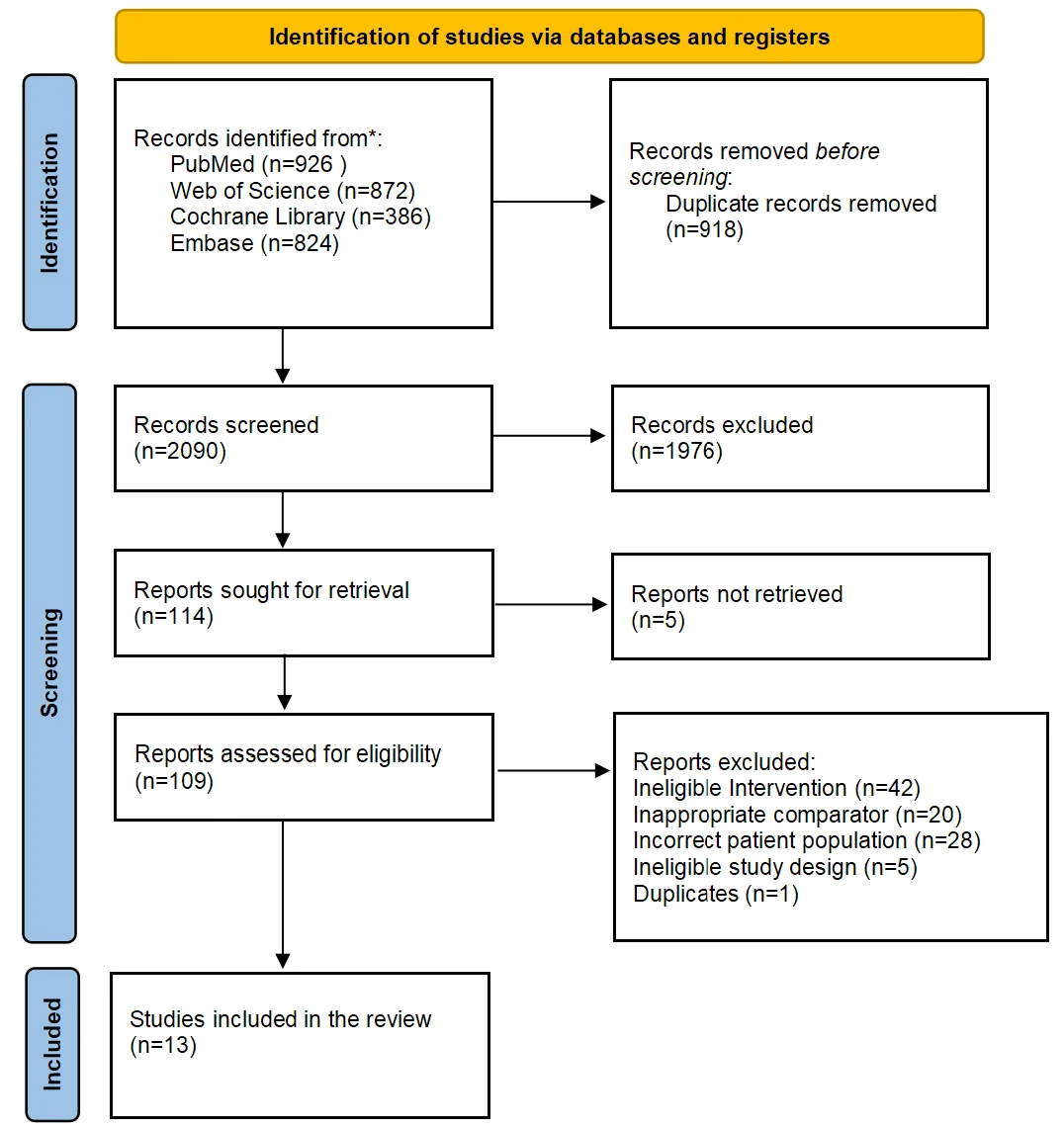
PRISMA (Preferred Reporting Items for Systematic Reviews and Meta-Analyses) flow diagram of the search and screening processes.

A total of 3008 records were identified through database searching, including PubMed (n=926), Web of Science (n=872), the Cochrane Library (n=386), and Embase (n=824). After removing 918 duplicate records, 2090 records were screened by title and abstract. Of these, 1976 records were excluded as irrelevant to the scope of this review. The full texts of 114 reports were sought for retrieval, of which 5 could not be obtained. Therefore, 109 reports were assessed for eligibility. During full-text review, 96 reports were excluded for the following reasons: ineligible intervention (n=42), inappropriate comparator (n=20), incorrect patient population (n=28), ineligible study design (n=5), and duplicate report (n=1). Ultimately, 13 studies were included in this systematic review and meta-analysis. The detailed study selection process is presented in Figure 1.

### Study Characteristics

The 13 randomized controlled trials included in this meta-analysis were published between 2012 and 2025 [18,39-50]. These studies were conducted across diverse geographical settings, including Turkey, China, Iran, Egypt, Saudi Arabia, the United States, Brazil, Belgium, and France. A total of 458 participants were enrolled across these studies, with individual sample sizes ranging from 6 to 62 children. All participants were children with hemiplegic CP, and their ages were limited to ≤12 years. The mean age of the included cohorts varied from 5.2 to 10.5 years. Most children were classified within levels 1 to 3 of the MACS and the GMFCS, indicating that the study population primarily consisted of children with mild to moderate functional impairments (Table S1 in Multimedia Appendix 3). The intervention protocols consistently compared VR-based intervention combined with conventional therapy against conventional therapy alone. The included interventions mainly used nonimmersive or screen-based VR or game-based systems, with commercial gaming systems such as Nintendo Wii being the most common. Session duration typically ranged from 30 to 60 minutes, and training frequency mostly ranged from 2 to 3 sessions per week over 2 to 12 weeks. Most interventions were delivered in clinical or hospital settings under professional supervision, while some studies used home-based platforms. No serious adverse events were reported across the included trials (Table S2 in Multimedia Appendix 3).

### Risk of Bias in Included Studies

#### Overview

**Figure 2:**
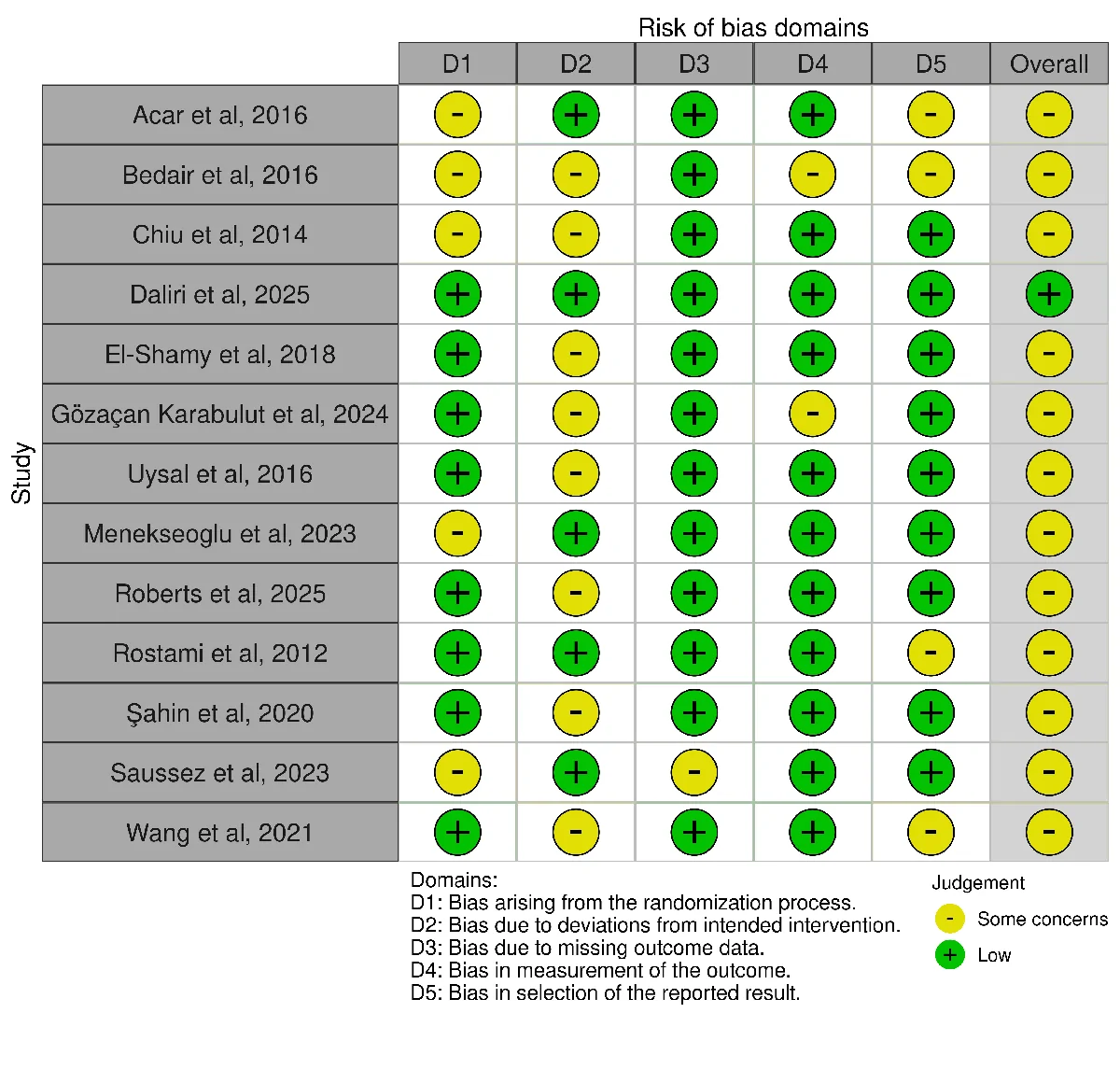
Risk-of-bias summary.

The included studies showed a generally moderate risk-of-bias profile. Most domains were rated as low risk in the majority of studies, particularly missing outcome data, outcome measurement, and selection of the reported result. Some concerns were mainly observed in the randomization process and deviations from intended interventions, reflecting incomplete reporting of allocation procedures or challenges in blinding participants and therapists in VR-based rehabilitation trials. The overall risk of bias was therefore judged as “some concerns” for most studies, with only 1 study rated as low overall risk (Figure 2 [18,39-50]).

#### Meta-Analysis

##### Upper-Limb Activity

**Figure 3:**
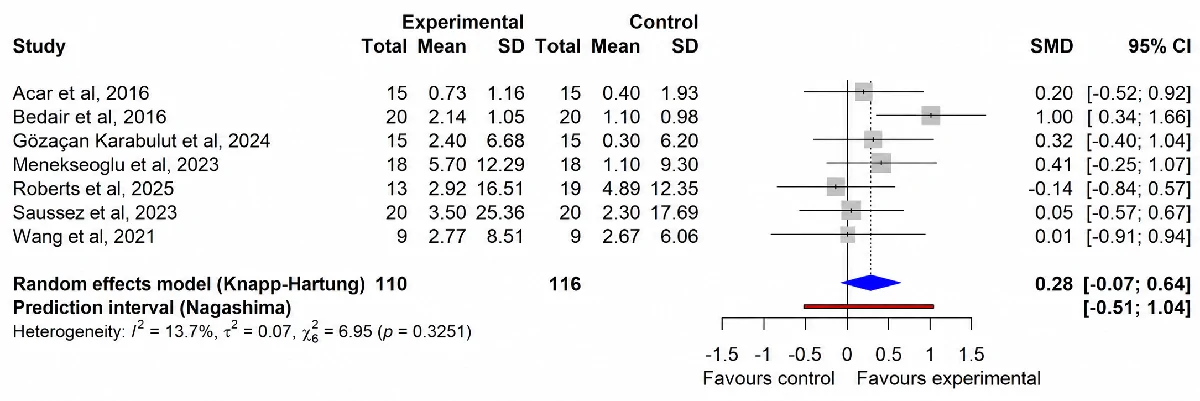
Forest plot comparing virtual reality–based intervention combined with conventional therapy versus conventional therapy alone for upper-limb activity.

**Figure 4:**
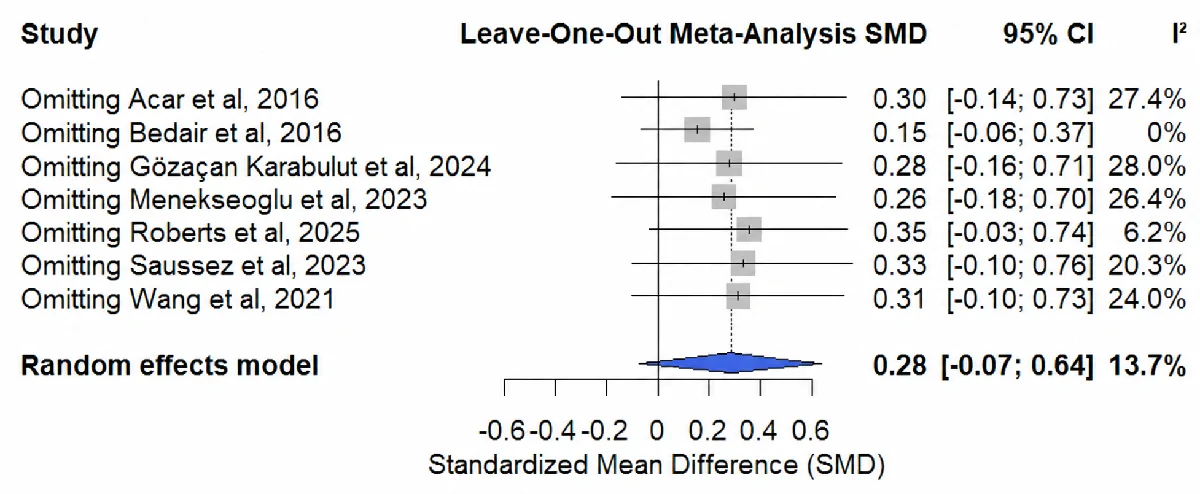
Sensitivity analysis plot comparing virtual reality–based intervention combined with conventional therapy versus conventional therapy alone for upper-limb activity.

Seven studies were included in the meta-analysis of upper-limb activity outcomes (Figure 3 [39,40,44-46,48,50]). Using a Knapp-Hartung–adjusted random-effects model, the pooled average effect numerically favored VR combined with conventional therapy; however, the difference was not statistically significant (SMD 0.28, 95% CI −0.07 to 0.64). As the 95% CI crossed the null value, the evidence remained uncertain for the average effect. Heterogeneity was low (Q=6.95; *P*=.33; *I*²=13.7%; τ²=0.07). The Nagashima-corrected 95% PI was wide and also crossed the null value (−0.51 to 1.04), indicating that effects in future comparable settings may range from no or potentially unfavorable effects to beneficial effects. Leave-one-out sensitivity analyses showed that the pooled point estimates remained broadly stable after sequentially omitting individual studies (SMD range 0.15-0.35), without materially changing the statistical interpretation (Figure 4 [39,40,44-46,48,50]).

##### Dexterity

**Figure 5:**
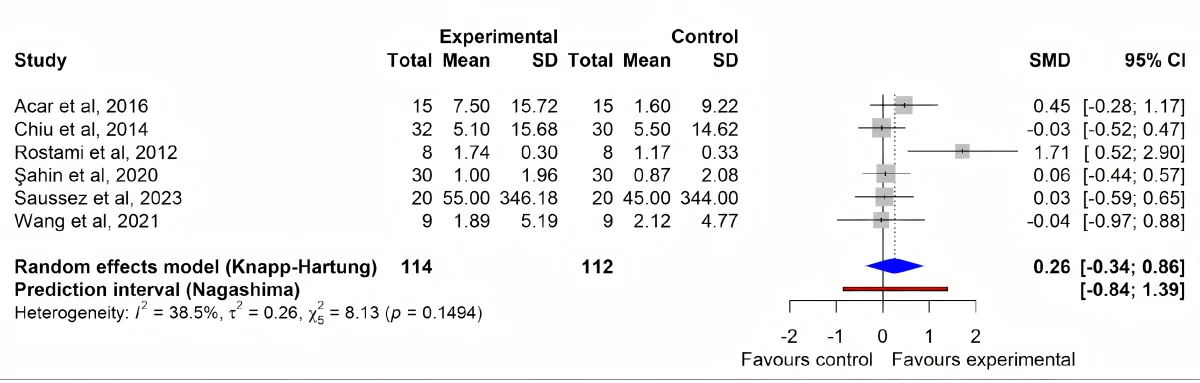
Forest plot comparing virtual reality–based intervention combined with conventional therapy versus conventional therapy alone for dexterity.

**Figure 6:**
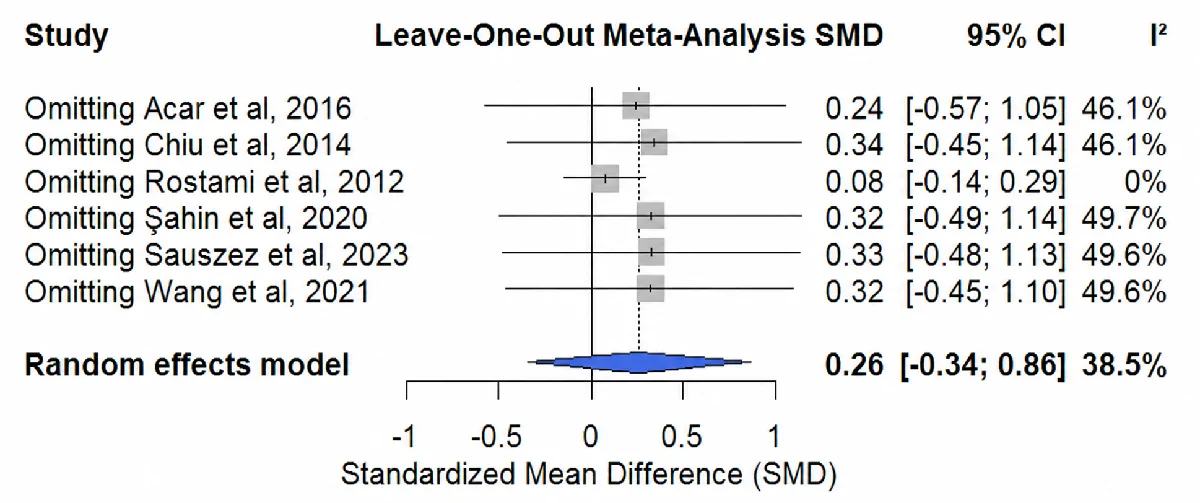
Sensitivity analysis plot comparing virtual reality–based intervention combined with conventional therapy versus conventional therapy alone for dexterity.

Six studies provided data for the dexterity outcome analysis (Figure 5 [39,41,47-50]). Using the Knapp-Hartung–adjusted random-effects model, the pooled estimate numerically favored VR combined with conventional therapy, but the difference was not statistically significant (SMD 0.26, 95% CI −0.34 to 0.86). The 95% CI crossed the null value, indicating uncertainty around the pooled effect. Moderate heterogeneity was observed across studies (Q=8.13; *P*=.15; *I*²=38.5%; τ²=0.26). The Nagashima-corrected 95% PI was wide and also crossed the null value (−0.84 to 1.39), suggesting that the true effect in future comparable studies may vary substantially. Leave-one-out sensitivity analysis showed that the pooled effects remained nonsignificant after sequentially omitting individual studies, with SMDs ranging from 0.08 to 0.34, indicating that no single study dominated the overall result (Figure 6 [39,41,47-50]).

##### Motor Control and Movement Quality

**Figure 7:**
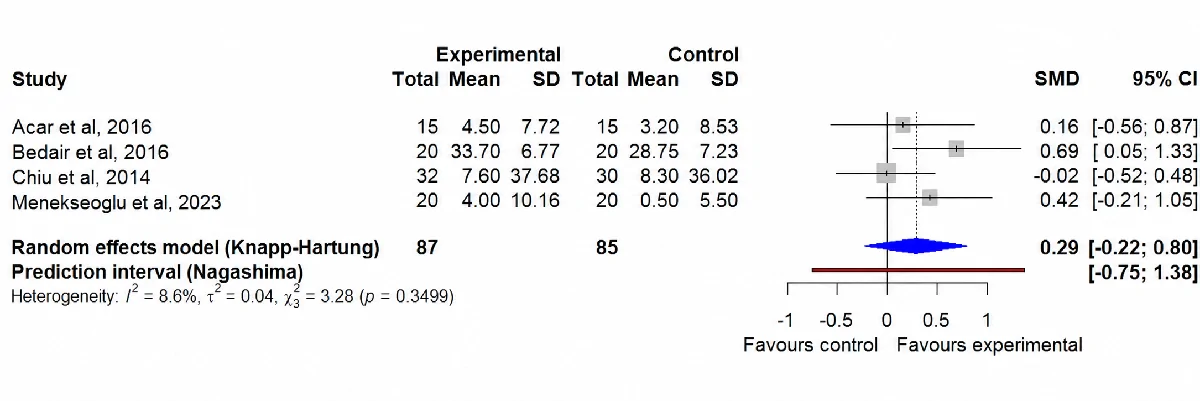
Forest plot comparing virtual reality–based intervention combined with conventional therapy versus conventional therapy alone for motor control and movement quality.

**Figure 8:**
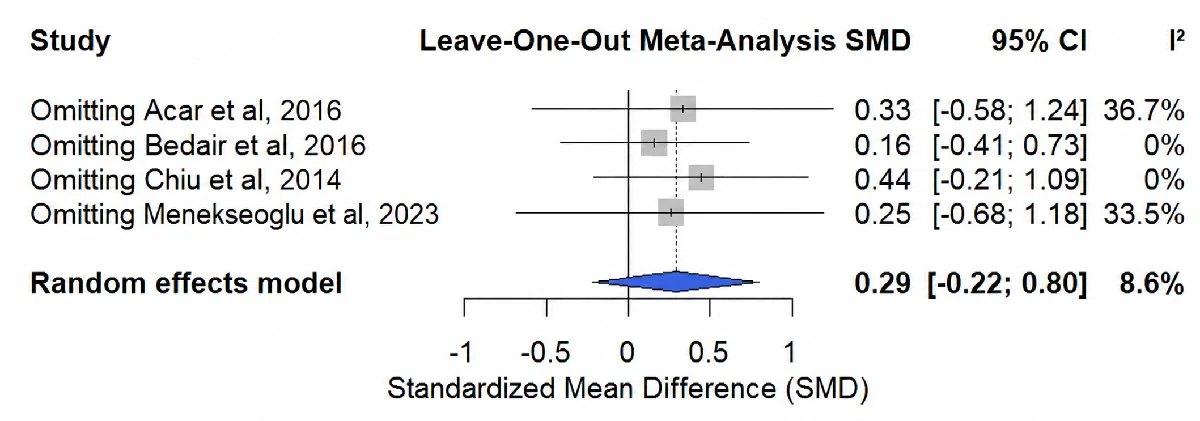
Sensitivity analysis comparing virtual reality–based intervention combined with conventional therapy versus conventional therapy alone for motor control and movement quality.

Four studies were included in the analysis of motor control and movement quality (Figure 7 [39-41,45]). Using the Knapp-Hartung–adjusted random-effects model, the pooled estimate numerically favored VR combined with conventional therapy, but the difference was not statistically significant (SMD 0.29, 95% CI −0.22 to 0.80). The 95% CI crossed the null value, suggesting uncertainty around the pooled effect size. Heterogeneity was low (Q=3.28; *P*=.35; *I*²=8.6%; τ²=0.04). The Nagashima-corrected 95% PI was wide and crossed the null value (−0.75 to 1.38), suggesting that the true effect in future comparable settings remains uncertain; however, this estimate should be interpreted cautiously because only 4 studies contributed to this outcome. Leave-one-out sensitivity analysis showed that the pooled effects remained nonsignificant after excluding each study in turn, with SMDs ranging from 0.16 to 0.44, indicating that the overall result was not driven by any single study (Figure 8 [39-41,45]).

##### Activities of Daily Living

**Figure 9:**
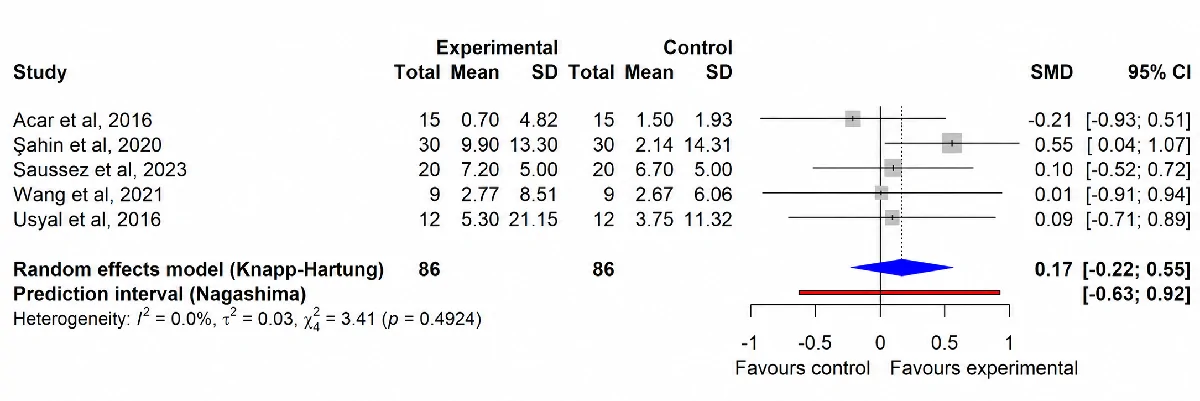
Forest plot comparing virtual reality–based intervention combined with conventional therapy versus conventional therapy alone for activities of daily living.

**Figure 10:**
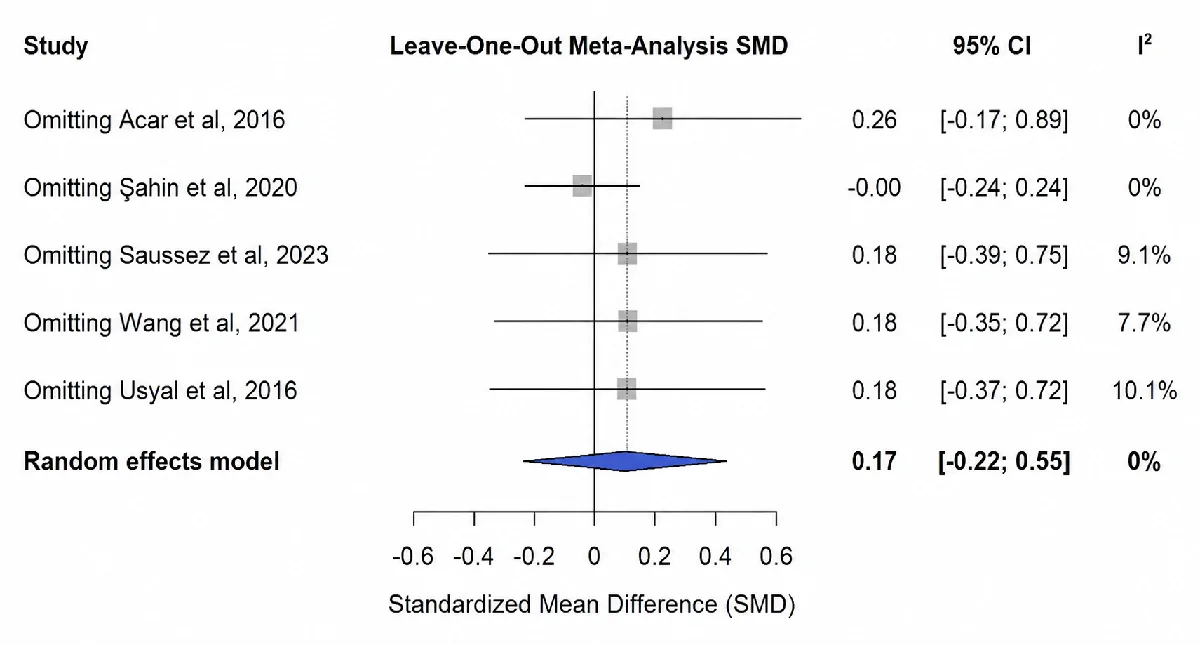
Sensitivity analysis plot comparing virtual reality–based intervention combined with conventional therapy versus conventional therapy alone for activities of daily living.

Five studies were included in the analysis of ADLs (Figure 9 [18,39,48-50]). Using the Knapp-Hartung random-effects model, the pooled estimate numerically favored VR combined with conventional therapy, but the difference was not statistically significant (SMD 0.17, 95% CI −0.22 to 0.55). The 95% CI crossed the null value, indicating uncertainty around the pooled effect. Heterogeneity was negligible across studies (Q=3.41, *P*=.49; *I*²=0.0%; τ²=0.03). The 95% Nagashima-corrected PI also crossed the null value (−0.63 to 0.92), suggesting that the true effect in future comparable studies remains uncertain. Leave-one-out sensitivity analysis showed that the pooled effects remained nonsignificant after sequential exclusion of individual studies, with SMDs ranging from approximately 0.00 to 0.26, indicating that no single study materially influenced the overall conclusion (Figure 10 [18,39,48-50]).

##### Grip Strength

**Figure 11:**
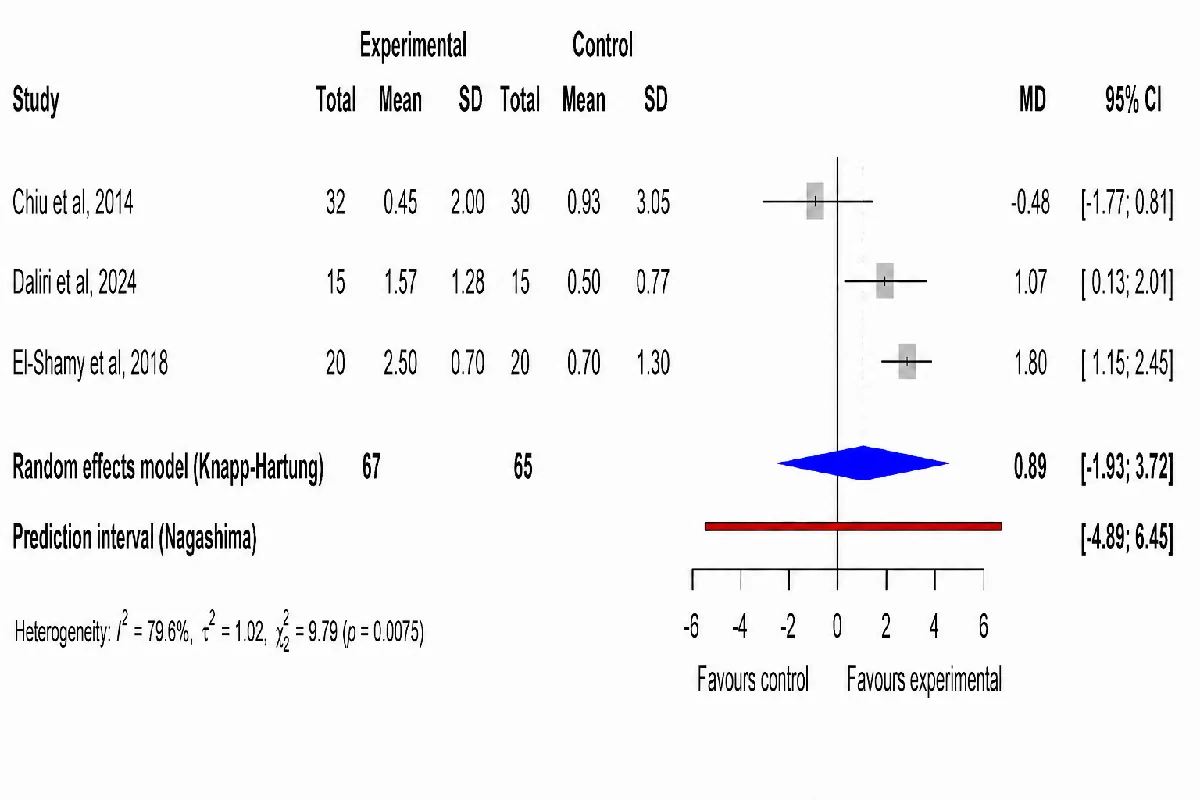
Forest plot comparing virtual reality–based intervention combined with conventional therapy versus conventional therapy alone for grip strength.

**Figure 12:**
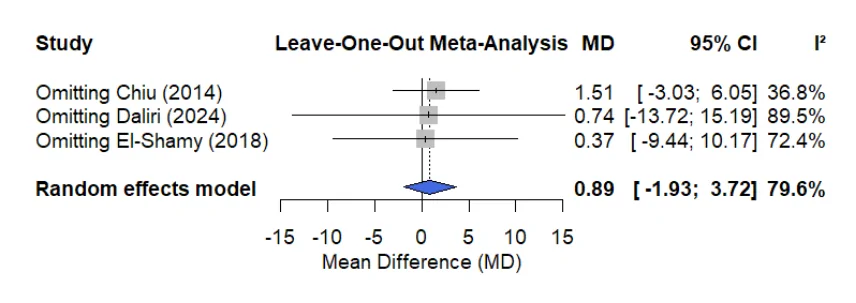
Sensitivity analysis plot comparing virtual reality–based intervention combined with conventional therapy versus conventional therapy alone for grip strength.

Three studies were included in the analysis of grip strength (Figure 11 [41-43]). Using the Knapp-Hartung random-effects model, the pooled estimate numerically favored VR combined with conventional therapy, but the difference was not statistically significant (MD 0.89, 95% CI −1.93 to 3.72). The 95% CI crossed the null value, indicating uncertainty around the pooled effect. In contrast to other outcomes, grip strength showed substantial heterogeneity (Q=9.79; *P*=.008; *I*²=79.6%; τ²=1.0157). Leave-one-out sensitivity analysis indicated that 1 study [41] was the main contributor to heterogeneity; excluding this study reduced *I*² from 79.6% to 36.8% (Figure 12 [41-43]). However, the pooled effect remained nonsignificant after removing any single trial, with MDs ranging from 0.37 to 1.51. Given the small number of studies and substantial heterogeneity, the findings for grip strength should be interpreted with caution.

#### Reporting Biases

Formal assessment of publication bias or small-study effects was not performed for any outcome because fewer than 10 studies were included in each outcome-specific meta-analysis.

#### Certainty of Evidence

The certainty of evidence was rated as low for upper-limb activity, dexterity, motor control and movement quality, and ADLs. The certainty for grip strength was rated as very low. The detailed GRADE table is provided in Table 1.

**Table 1:** Summary of findings and certainty of evidence assessed using the Grading of Recommendations Assessment, Development, and Evaluation approach.

Certainty assessment									Patients, n			Effect			Certainty		Importance
Topic and study design		Studies, n	Risk of bias	Inconsistency	Indirectness	Imprecision	Other considerations	VR^a^+CT^b^		CT	Relative (95% CI)		Absolute (95% CI)				
**Upper limb activity**																	
	Randomized trials	7	Serious	Not serious	Not serious	Serious	None	110		116	—^c^		SMD^d^ *0.28 SD higher* (0.07 lower to 0.64 higher)	⊕⊕○○ Low		Critical	
**Dexterity**																	
	Randomized trials	6	Serious	Not serious	Not serious	Serious	None	114		112	—		SMD *0.26 SD higher* (0.34 lower to 0.86 higher)	⊕⊕○○ Low		Critical	
**Motor control and movement quality**																	
	Randomized trials	4	Serious	Not serious	Not serious	Serious	None	87		85	—		SMD *0.29 SD higher* (0.22 lower to 0.8 higher)	⊕⊕○○ Low		Critical	
**ADL^e^**																	
	Randomized trials	5	Serious	Not serious	Not serious	Serious	None	86		86	—		SMD *0.17 SD higher* (0.22 lower to 0.55 higher)	⊕⊕○○ Low		Important	
**Grip strength**																	
	Randomized trials	3	Serious	Serious	Not serious	Serious	None	67		65	—		MD^f^ *0.89kg MD higher* (1.93kg lower to 3.72kg higher)	⊕○○○ Very low		Important	

^a^VR: virtual reality.

^b^CT: conventional therapy.

^c^Not applicable.

^d^SMD: standardized mean difference. SMDs are expressed in SD units.

^e^ADL: activity of daily living.

^f^MD: mean difference.

## Discussion

### Principal Findings

This systematic review and meta-analysis aimed to clarify whether adding VR-based intervention to conventional therapy provides additional benefits for upper-limb rehabilitation in children with hemiplegic CP. By focusing on this specific population, restricting the comparator to conventional therapy alone, and separating upper-limb outcomes into clinically distinct domains, this review provides a more conservative and domain-specific estimate of the adjunctive effect of VR-based intervention. Overall, the pooled estimates were directionally favorable to VR combined with conventional therapy across upper-limb activity, dexterity, motor control and movement quality, ADLs, and grip strength. However, no outcome reached statistical significance, and the certainty of evidence ranged from low to very low. Therefore, the current randomized evidence remains insufficient to confirm robust additional benefits of adding VR-based intervention to conventional therapy.

These findings should be interpreted cautiously in relation to effect uncertainty, heterogeneity, risk of bias, and certainty of evidence. Although the point estimates generally favored the combined intervention, the CIs indicated uncertainty around the average pooled effects, while the PIs suggested that the true effects may vary across comparable future settings [35]. Heterogeneity was generally low to moderate for most outcomes, but these estimates were based on few studies; grip strength showed substantial heterogeneity and therefore requires greater caution [30,35]. In addition, most included trials had some concerns in the RoB 2 assessment [26]. Taken together, these issues indicate that the favorable direction of the pooled estimates should be interpreted as hypothesis generating rather than confirmatory evidence of clinical effectiveness.

Several factors may explain this uncertainty. First, many included interventions used commercially available game-based systems rather than rehabilitation-specific VR platforms. They may not provide sufficiently individualized, impairment-specific, or progressively adapted upper-limb training [51]. From a motor learning perspective, simple repetition of limb movements may be insufficient; meaningful improvement is more likely when practice is goal directed, skill based, feedback guided, and progressively challenging [21,52]. Second, most interventions were delivered through nonimmersive or screen-based VR systems. A key feature of VR rehabilitation is interaction with a multidimensional and multisensory virtual environment [53]. Nonimmersive systems may be feasible and accessible, but limited sensory richness, embodiment, and tactile or haptic feedback may reduce their capacity to target fine hand use and movement quality [53-55]. A previous study suggested that integrating visual-tactile interfaces with motion tracking may enhance VR-mediated hand rehabilitation training [56]. Third, intervention dose varied across trials, including program duration, training frequency, session length, and total practice time, which may have contributed to variability in the observed effects.

Although the pooled effects were small and statistically nonsignificant, the generally favorable direction of effect suggests that well-designed VR protocols may still have potential as an adjunctive rehabilitation strategy. From a motor learning perspective, VR-based systems may support rehabilitation by providing repetitive, task-oriented practice; real-time feedback; and enriched visuomotor engagement [2,4,57]. These features may enhance motivation and provide a more engaging training context for children [51]. However, these mechanisms remain speculative because neurophysiological outcomes were not directly assessed in the included trials.

The findings of the present review are not fully consistent with previous meta-analytic evidence on VR-based rehabilitation in children with CP. Burin-Chu et al [7] reported a significant benefit of VR combined with conventional therapy on upper-limb activity, whereas the present review found only a small and statistically nonsignificant effect. This discrepancy may be partly attributable to differences in the included populations: the former review included children and adolescents with unilateral or bilateral CP, whereas the present review focused specifically on children with hemiplegic CP, which may have influenced the pooled effect estimate. Ibrahim et al [12] more closely matched the present review in terms of population by focusing on children with hemiplegic CP and reported significant improvements in hand function and upper extremity skill quality. However, its intervention and comparator conditions were broader, including comparisons of VR with no intervention, conventional therapy, or alternative rehabilitation approaches, whereas the present review specifically estimated the additional effect of VR combined with conventional therapy compared with conventional therapy alone. In addition, unlike previous reviews, the present review further separated upper-limb–related outcomes into more specific domains, which reduced the number of studies contributing to each analysis. This may have decreased statistical precision and resulted in more conservative pooled effect estimates.

Sensitivity analysis indicated that the grip strength finding lacked robustness and was heavily influenced by 1 study [41]. Further scrutiny of the original report suggested that this disproportionate influence was likely attributable to the baseline imbalance in grip strength between the groups. Therefore, these results necessitate a cautious interpretation.

### Strengths and Limitations

The main strength of this review is its clinically specific design. By restricting the population to children with hemiplegic CP and limiting the comparison to VR-based intervention combined with conventional therapy versus conventional therapy alone, this review reduced clinical ambiguity and provided a more direct estimate of the adjunctive effect of VR-based training in conventional upper-limb rehabilitation. In addition, upper-limb–related outcomes were classified according to the ICF framework [58] and the primary constructs measured by individual instruments, which improved clinical interpretability across outcome domains. The use of random-effects models with the HKSJ adjustment, PIs, GRADE assessment, and leave-one-out sensitivity analyses also supported a more conservative interpretation of the evidence [35,38].

However, several limitations should be considered. First, the number of included studies and participants was limited, and each outcome-specific analysis included only a small number of trials. This reduced statistical precision and limited the ability to determine whether small or moderate effects were present. Second, the included trials varied in VR device type, intervention content, session duration, training frequency, total intervention period, treatment setting, and supervision status, which limited the ability to identify which intervention characteristics may be associated with better outcomes. Third, most included studies had some concerns in the RoB 2 assessment, which reduced confidence in the robustness of the pooled estimates. Fourth, we did not contact study authors to obtain missing or unclear data, and outcomes with insufficient extractable data could not be included in the quantitative synthesis. Finally, because fewer than 10 studies contributed to each outcome, formal assessment of publication bias and small-study effects was not feasible [59]. These limitations contributed to the low to very low certainty of evidence and should be considered when interpreting the findings.

### Clinical Implications

Clinically, VR-based intervention may be considered a potentially engaging adjunct to conventional upper-limb rehabilitation for children with hemiplegic CP. Its potential value lies in providing interactive, feedback-rich, and repetitive practice that may improve motivation and task engagement [4,60]. However, given the low to very low certainty of evidence and the absence of statistically significant effects across outcome domains, the present findings do not support using VR as a replacement for conventional therapy or as a definitively effective intervention for improving upper-limb function. When VR is used in practice, it should be embedded within a structured rehabilitation program and aligned with the child’s functional goals [61,62]. VR tasks should be task-specific, progressively adapted, and supervised by therapists where possible [63], with attention to intervention dose; training intensity; and clinically meaningful outcomes, such as affected upper-limb use, bimanual coordination, object manipulation, and ADLs. Future trials should use larger samples, clearer reporting of VR characteristics and intervention dose, and standardized outcome measures to determine whether specific VR protocols can produce clinically meaningful upper-limb gains.

### Conclusions

This review provides a focused and conservative synthesis of randomized evidence on VR-based intervention combined with conventional therapy for upper-limb rehabilitation in children with hemiplegic CP. The current evidence does not confirm robust additional benefits of adding VR to conventional therapy, and the findings should be interpreted cautiously because the certainty of evidence was low to very low. Nevertheless, this review has broader clinical and practical implications. For clinicians, VR may be considered a potentially engaging adjunct to conventional therapy, but it should not be used as a replacement for therapist-led rehabilitation or presented as a definitively effective intervention. For the development and implementation of digital rehabilitation technologies, the findings suggest that future VR systems should move beyond general game-based practice toward rehabilitation-specific, task-oriented, progressively adapted, and clinically measurable training protocols. Future trials should therefore use adequately powered randomized designs, standardized upper-limb outcome domains, clearly reported VR characteristics and intervention dose, and longer follow-up to determine whether specific VR approaches can produce clinically meaningful functional gains.

## Data Availability

The data extracted from the included studies and used for the meta-analyses are available from the corresponding author upon reasonable request.

## Appendix

Multimedia Appendix 1 Search strategy.

Multimedia Appendix 2 Outcome instrument classification.

Multimedia Appendix 3 Characteristics of the included studies and interventions.

Multimedia Appendix 4 PRISMA 2020 checklist.

Multimedia Appendix 5 PRISMA-S 2020 checklist.

## Abbreviations

ADL: activity of daily living
CP: cerebral palsy
GMFCS: Gross Motor Functional Classification System
GRADE: Grading of Recommendations Assessment, Development, and Evaluation
HKSJ: Hartung-Knapp-Sidik-Jonkman
ICF: International Classification of Functioning, Disability and Health
MACS: Manual Ability Classification System
MD: mean difference
PI: prediction interval
PRESS: Peer Review of Electronic Search Strategies
PRISMA-S: Preferred Reporting Items for Systematic Reviews and Meta-Analyses literature search extension
RoB 2: revised Cochrane risk-of-bias 2 tool
SMD: standardized mean difference
VR: virtual reality
