# Supplementary material for: Effects of Virtual Reality–Based Intervention Combined With Conventional Therapy on Upper-Limb Function in Children With Hemiplegic Cerebral Palsy: Systematic Review and Meta-Analysis of Randomized Controlled Trials
Source: JMIR Serious Games. 2026 Aug 26;14:e98694. doi: 10.2196/98694 (PMC13559153; doi:10.2196/98694)
Supplement: Supplementary file 1 [file games_v14i1e98694_app1.docx]

**Table. Search strategy**

**Pubmed**

| #1 | "Cerebral Palsy"[Mesh] OR "cerebral palsy"[Title/Abstract] OR "hemiplegic cerebral palsy"[Title/Abstract] OR "unilateral cerebral palsy"[Title/Abstract] OR "spastic hemiplegia"[Title/Abstract] OR "hemiplegic CP"[Title/Abstract] OR "unilateral CP"[Title/Abstract] OR hemiplegia[Title/Abstract] OR hemiparesis[Title/Abstract] |
| --- | --- |
| #2 | "Virtual Reality"[Mesh] OR "virtual reality"[Title/Abstract] OR VR[Title/Abstract] OR "virtual environment"[Title/Abstract] OR "virtual environments"[Title/Abstract] OR "virtual rehabilitation"[Title/Abstract] OR "virtual training"[Title/Abstract] OR "computer-assisted therapy"[Title/Abstract] OR "computer assisted therapy"[Title/Abstract] OR "video game"[Title/Abstract] OR "video games"[Title/Abstract] OR videogame[Title/Abstract] OR videogames[Title/Abstract] OR exergam*[Title/Abstract] OR "active video game"[Title/Abstract] OR "active video games"[Title/Abstract] OR "interactive game"[Title/Abstract] OR "interactive games"[Title/Abstract] OR "interactive gaming"[Title/Abstract] OR "motion-controlled game"[Title/Abstract] OR "motion-controlled games"[Title/Abstract] OR "motion controlled game"[Title/Abstract] OR "motion controlled games"[Title/Abstract] OR "Nintendo Wii"[Title/Abstract] OR Wii[Title/Abstract] OR "Wii Fit"[Title/Abstract] OR "Xbox Kinect"[Title/Abstract] OR Kinect[Title/Abstract] OR Xbox[Title/Abstract] OR PlayStation[Title/Abstract] OR "PlayStation EyeToy"[Title/Abstract] OR EyeToy[Title/Abstract] OR "head-mounted display"[Title/Abstract] OR "head mounted display"[Title/Abstract] OR HMD[Title/Abstract] |
| #3 | "Upper Extremity"[Mesh] OR "upper extremity"[Title/Abstract] OR "upper limb"[Title/Abstract] OR "upper limbs"[Title/Abstract] OR arm[Title/Abstract] OR arms[Title/Abstract] OR hand[Title/Abstract] OR hands[Title/Abstract] OR "hand function"[Title/Abstract] OR "upper limb function"[Title/Abstract] OR "upper extremity function"[Title/Abstract] OR "upper limb activity"[Title/Abstract] OR "upper extremity activity"[Title/Abstract] OR "manual ability"[Title/Abstract] OR "bimanual performance"[Title/Abstract] OR "fine motor"[Title/Abstract] OR "fine motor skill"[Title/Abstract] OR "fine motor skills"[Title/Abstract] OR dexterity[Title/Abstract] OR "manual dexterity"[Title/Abstract] OR "motor function"[Title/Abstract] OR "motor control"[Title/Abstract] OR "selective motor control"[Title/Abstract] OR "activities of daily living"[Title/Abstract] OR ADL[Title/Abstract] OR "functional independence"[Title/Abstract] OR "grip strength"[Title/Abstract] OR "Assisting Hand Assessment"[Title/Abstract] OR AHA[Title/Abstract] OR "ABILHAND-Kids"[Title/Abstract] OR ABILHAND[Title/Abstract] OR "Jebsen-Taylor"[Title/Abstract] OR JTHFT[Title/Abstract] OR JTTHF[Title/Abstract] OR "Box and Block Test"[Title/Abstract] OR BBT[Title/Abstract] OR "Nine-Hole Peg Test"[Title/Abstract] OR "9-Hole Peg Test"[Title/Abstract] OR 9HPT[Title/Abstract] OR "Quality of Upper Extremity Skills Test"[Title/Abstract] OR QUEST[Title/Abstract] OR WeeFIM[Title/Abstract] OR PEDI[Title/Abstract] |
| #4 | "Child"[Mesh] OR child[Title/Abstract] OR children[Title/Abstract] OR pediatric[Title/Abstract] OR paediatric[Title/Abstract] OR infant[Title/Abstract] OR preschool[Title/Abstract] OR "school-aged"[Title/Abstract] OR "school aged"[Title/Abstract] |
| #5 | randomized controlled trial[Publication Type] OR controlled clinical trial[Publication Type] OR randomized[Title/Abstract] OR randomised[Title/Abstract] OR randomly[Title/Abstract] OR trial[Title/Abstract] OR "controlled trial"[Title/Abstract] OR "clinical trial"[Title/Abstract] OR RCT[Title/Abstract] |
| #6 | animals[Mesh] NOT humans[Mesh] |
| #7 | #1 AND #2 AND #3 AND #4 AND #5 NOT #6 |

**WOS**

| #1 | TS=("cerebral pals*" OR "hemiplegic cerebral pals*" OR "unilateral cerebral pals*" OR "spastic hemipleg*" OR "unilateral CP" OR "hemiplegic CP" OR hemiplegi* OR hemipares*) |
| --- | --- |
| #2 | TS=("virtual realit*" OR "virtual environment*" OR "virtual rehabilitation" OR "virtual training" OR "virtual therap*" OR "computer-assisted therap*" OR "computer assisted therap*" OR exergam* OR "active video game*" OR "interactive video game*" OR "interactive game*" OR "motion-controlled game*" OR "motion controlled game*" OR "video game*" OR videogame* OR "serious game*" OR "Nintendo Wii" OR Wii OR "Wii Fit" OR Kinect OR "Xbox Kinect" OR Xbox OR PlayStation OR "PlayStation EyeToy" OR EyeToy OR "head-mounted display*" OR "head mounted display*" OR HMD) |
| #3 | TS=("upper limb*" OR "upper extremit*" OR arm OR arms OR hand OR hands OR "hand function" OR "manual ability" OR "bimanual performance" OR dexter* OR "manual dexter*" OR "fine motor" OR "motor function" OR "motor control" OR "selective motor control" OR "activities of daily living" OR ADL OR "functional independence" OR "grip strength") |
| #4 | TS=(child* OR pediatric* OR paediatric* OR preschool* OR "school-aged" OR "school aged" OR adolescen*) |
| #5 | TS=(random* OR trial* OR "controlled trial*" OR "clinical trial*" OR RCT OR "randomized controlled trial*" OR "randomised controlled trial*") |
| #6 | #1 AND #2 AND #3 AND #4 AND #5 NOT #6 |

**Embae**

| #1 | 'cerebral palsy'/exp OR 'hemiplegia'/exp OR 'hemiparesis'/exp OR 'cerebral pals*':ti,ab,kw OR 'hemiplegic cerebral pals*':ti,ab,kw OR 'unilateral cerebral pals*':ti,ab,kw OR 'spastic hemipleg*':ti,ab,kw OR 'hemiplegic cp':ti,ab,kw OR 'unilateral cp':ti,ab,kw OR hemiplegi*:ti,ab,kw OR hemipares*:ti,ab,kw |
| --- | --- |
| #2 | 'virtual reality'/exp OR 'video game'/exp OR 'computer game'/exp OR 'virtual realit*':ti,ab,kw OR 'virtual environment*':ti,ab,kw OR 'virtual rehabilitation':ti,ab,kw OR 'virtual training':ti,ab,kw OR 'virtual therap*':ti,ab,kw OR 'computer assisted therap*':ti,ab,kw OR 'computer-assisted therap*':ti,ab,kw OR exergam*:ti,ab,kw OR 'active video game*':ti,ab,kw OR 'interactive video game*':ti,ab,kw OR 'interactive game*':ti,ab,kw OR 'interactive gaming':ti,ab,kw OR 'motion controlled game*':ti,ab,kw OR 'motion-controlled game*':ti,ab,kw OR 'serious game*':ti,ab,kw OR 'video game*':ti,ab,kw OR videogame*:ti,ab,kw OR 'nintendo wii':ti,ab,kw OR wii:ti,ab,kw OR 'wii fit':ti,ab,kw OR kinect:ti,ab,kw OR 'xbox kinect':ti,ab,kw OR xbox:ti,ab,kw OR playstation:ti,ab,kw OR eyetoy:ti,ab,kw OR 'head mounted display*':ti,ab,kw OR 'head-mounted display*':ti,ab,kw OR hmd:ti,ab,kw |
| #3 | 'child'/exp OR 'preschool child'/exp OR 'school child'/exp OR 'pediatrics'/exp OR child*:ti,ab,kw OR pediatric*:ti,ab,kw OR paediatric*:ti,ab,kw OR preschool*:ti,ab,kw OR 'school aged':ti,ab,kw OR 'school-aged':ti,ab,kw OR adolescen*:ti,ab,kw |
| #4 | 'randomized controlled trial'/exp OR 'controlled clinical trial'/exp OR 'clinical trial'/exp OR random*:ti,ab,kw OR trial*:ti,ab,kw OR 'controlled trial*':ti,ab,kw OR 'clinical trial*':ti,ab,kw OR rct:ti,ab,kw |
| #5 | 'animal'/exp NOT 'human'/exp |
| #6 | #1 AND #2 AND #3 AND #4 NOT #5 |

**Cochrane**

| #1 | MeSH descriptor: [Cerebral Palsy] explode all trees |
| --- | --- |
| #2 | MeSH descriptor: [Hemiplegia] explode all trees |
| #3 | ("cerebral pals*" OR "hemiplegic cerebral pals*" OR "unilateral cerebral pals*" OR "spastic hemipleg*" OR "hemiplegic CP" OR "unilateral CP" OR hemiplegi* OR hemipares*):ti,ab,kw |
| #4 | #1 OR #2 OR #3 |
| #5 | MeSH descriptor: [Virtual Reality] explode all trees |
| #6 | ("virtual realit*" OR "virtual environment*" OR "virtual rehabilitation" OR "virtual training" OR "virtual therap*" OR "computer assisted therap*" OR "computer-assisted therap*" OR exergam* OR "active video game*" OR "interactive video game*" OR "interactive game*" OR "interactive gaming" OR "motion controlled game*" OR "motion-controlled game*" OR "serious game*" OR "video game*" OR videogame* OR "Nintendo Wii" OR Wii OR "Wii Fit" OR Kinect OR "Xbox Kinect" OR Xbox OR PlayStation OR EyeToy OR "head mounted display*" OR "head-mounted display*" OR HMD):ti,ab,kw |
| #7 | #5 OR #6 |
| #8 | MeSH descriptor: [Child] explode all trees |
| #9 | MeSH descriptor: [Pediatrics] explode all trees |
| #10 | (child* OR pediatric* OR paediatric* OR preschool* OR "school aged" OR "school-aged" OR adolescen*):ti,ab,kw |
| #11 | #8 OR #9 OR #10 |
| #12 | (random* OR trial* OR "controlled trial*" OR "clinical trial*" OR RCT):ti,ab,kw |
| #12 | #4 AND #7 AND #11 AND #12 |
