# Supplementary material for: Effects of Virtual Reality–Based Intervention Combined With Conventional Therapy on Upper-Limb Function in Children With Hemiplegic Cerebral Palsy: Systematic Review and Meta-Analysis of Randomized Controlled Trials
Source: JMIR Serious Games. 2026 Aug 26;14:e98694. doi: 10.2196/98694 (PMC13559153; doi:10.2196/98694)
Supplement: Supplementary file 2 [file games_v14i1e98694_app2.docx]

| **Instrument / measure** | **Main construct assessed** | **Classified outcome domain** | **Rationale for classification** |
| --- | --- | --- | --- |
| AHA | Use of the affected hand in bimanual activities | Upper limb activity | Assesses actual affected-hand use during functional bimanual tasks |
| ABILHAND-Kids | Manual ability in daily bimanual activities | Upper limb activity | Reflects activity-level manual performance rather than isolated hand speed or strength |
| JTHFT / JTTHF | Timed hand task performance | Dexterity | Measures speed and efficiency of hand manipulation during standardized tasks |
| BBT | Object transfer/manual dexterity | Dexterity | Assesses rapid object manipulation and hand dexterity |
| BOTMP / BOT-2 Speed and Dexterity | Speed and dexterity of hand manipulation | Dexterity | Primarily evaluates fine motor speed, precision, and manual efficiency |
| PDMS-2 Object Manipulation | Object manipulation and hand-eye coordination | Dexterity | Mainly reflects object-handling performance rather than global ADL independence |
| QUEST | Quality of upper-extremity movement | Motor control and movement quality | Assesses dissociated movement, grasp, weight bearing, protective extension, and qualitative movement performance |
| FUS | Functional/qualitative use of the affected limb | Motor control and movement quality | Reflects quality of affected-limb use rather than only task completion or independence |
| WeeFIM | Functional independence in daily activities | Activities of daily living | Measures independence in everyday functional tasks |
| PEDI | Pediatric functional ability and daily task performance | Activities of daily living | Assesses functional capability and independence in daily activities |
| Grip strength | Maximal voluntary handgrip force | Grip strength | Direct measure of handgrip force |

**Table. Classification of outcome instruments by primary measurement construct**
