# Supplementary material for: Effects of Virtual Reality–Based Intervention Combined With Conventional Therapy on Upper-Limb Function in Children With Hemiplegic Cerebral Palsy: Systematic Review and Meta-Analysis of Randomized Controlled Trials
Source: JMIR Serious Games. 2026 Aug 26;14:e98694. doi: 10.2196/98694 (PMC13559153; doi:10.2196/98694)
Supplement: Supplementary file 3 [file games_v14i1e98694_app3.docx]

**Table 1.Basic characteristics**

| **First author (year)** | **Country** | **Design** | **CP Type** | **Age (year)** | **Gender** | **Classification** | **Sample size** | **Outcomes** |
| --- | --- | --- | --- | --- | --- | --- | --- | --- |
| Acar (2016) | Turkey | RCT | Hemiparesis | IG: 9.53 ± 3.04, CG: 9.73 ± 2.86 | F: 16, M: 14 | MACS: Ⅰ-Ⅲ GMFCS: Ⅰ-Ⅱ | IG: 15, CG: 15 | ABILHAND-Kids,JTHFT,QUEST,WeeFIM |
| Bedair | Egypt | RCT | Hemiparesis | IG: 7.05 ± 0.99, CG: 7.25 ± 0.96 | F: 17, M: 23 | MACS: Ⅰ-Ⅱ GMFCS: Ⅰ-Ⅱ | IG: 20, CG: 20 | ABILHAND-Kids，PDMS-2(Object Manipulation) |
| Chiu et al., 2014 | China | RCT | Hemiparesis | IG: 9.4 ± 1.9, CG: 9.5 ± 1.9 | M: 28 | MACS: Ⅰ-Ⅴ GMFCS: Ⅰ-Ⅴ | IG: 32, CG: 30 | JIHFT,FUS,Grip strength |
| Daliri M (2025) | Iran | RCT | Hemiparesis | IG: 7.2 ± 1.2, CG: 7.6 ± 1.18 | F: 9, M: 6 | MACS: Ⅰ-Ⅲ GMFCS: Ⅰ-Ⅱ | IG: 15, CG: 15 | Grip strength |
| El-Shamy (2018) | Egypt, Saudi Arabia | RCT | Hemiparesis | IG: 9.5±1.2, CG: 9.8±1.4 | F: 14, M: 26 | MACS: Ⅰ-Ⅲ | IG: 20, CG: 20 | Grip strength |
| Gözaçan Karabulut (2024) | Turkey | RCT | Hemiparesis | IG: 9.2 ± 3.8, CG: 8.3 ± 4.1 | NA | MACS: Ⅰ-Ⅲ GMFCS: Ⅰ-Ⅲ | IG: 15, CG: 15 | ABILHAND-Kids |
| Uysal（2016） | Turkey | RCT | Hemiparesis | IG: 9.13 ± 2.57, CG: 10.11 ± 2.62 | F: 14, M: 10 | MACS: Ⅰ-Ⅱ GMFCS: Ⅰ-Ⅲ | IG: 12, CG: 12 | PEDI |
| Menekseoglu(2023) | Turkey | RCT | Hemiparesis | IG: 8.2±1.8, CG: 8.3±1.4 | F: 17, M: 19 | MACS: Ⅰ-Ⅱ GMFCS: Ⅰ-Ⅲ | IG: 18, CG: 18 | ABILHAND-Kids,AHA,QUEST |
| Roberts(2025) | United States | RCT | Hemiparesis | IG: 10.25, CG: 8.75 | F: 19, M: 13 | MACS: Ⅰ-Ⅲ GMFCS: Ⅰ-Ⅱ | IG: 13, CG: 19 | AHA |
| Rostami (2012) | Iran | RCT | Hemiparesis | 8.04 ± 0.29 | F: 18, M: 14 | MAS:Ⅰ-Ⅱ | IG: 8, CG: 8, | BOTMP-Speed & Dexterity |
| Şahin (2020) | Turkey | RCT | Hemiparesis | IG: 10.5 ± 3.62, CG: 10.06 ± 3.24 | F: 23, M: 37 | MACS: Ⅰ-Ⅱ GMFCS: Ⅰ-Ⅲ | IG: 30, CG: 30 | BOTMP-Speed & Dexterity,WeeFIM |
| Saussez (2023) | Belgium, France | RCT | Hemiparesis | IG: 9.0 ± 3.1, CG: 9.1 ± 2.9 | F: 20, M: 20, | MACS: Ⅰ-Ⅲ GMFCS: Ⅰ-Ⅱ | IG: 20, CG: 20 | ABILHAND-Kids,AHA,BBT,JTTHF,PEDI |
| Wang (2021) | China | RCT | Hemiparesis | 8.56 ± 2.09 | F: 11, M: 7 | MACS: Ⅰ-Ⅲ | IG: 9, CG: 9 | ABILHAND-Kids,BOT-2,WeeFIM |

IG:Intervention group; CG:Controlled group; F:Female;M:Male; MACS:Manual Ability Classification System; GMFCS:Gross Motor Function Classification System;

JTHFT:Jebsen-Taylor Hand Function Test; QUEST:Quality of Upper Extremity Skills Test; WeeFIM:Functional Independence Measure for Children; PDMS:Peabody Developmental Motor Scales; FUS:Functional Use Survey;Nine-Hole Peg Test; AHA:Assisting Hand Assessment; BBT:Box and Block Test; PEDI:Pediatric Evaluation of Disability Inventory; BOTMP:Bruininks-Oseretsky Test of Motor Proficiency; BOT:Bruininks-Oseretsky Test of Motor Proficiency

**Table 2. Intervention Characteristics**

| **Study** | **Intervention** | **Comparision** | **Device** | **Virtual scenario** | **VR type** | **Time** | **Frequence** | **Period** | **Setting** | **Supervision** | **Adverse events** |
| --- | --- | --- | --- | --- | --- | --- | --- | --- | --- | --- | --- |
| Acar (2016) | VRT + CT | CT | Nintendo Wii^®^ | Tennis,Baseball,Boxing | Non-immersive | 45 min | 2 sessions per week | 6 weeks | Clinic | Yes | NA |
| Bedair | VR + CT | CT | Microsoft Xbox, Kinect | Tennis, bowling, golf, space pop, bubbles, boat driving | Non-immersive | 30 min | 3 sessions per week | 12 weeks | Clinic | Yes | No serious adverse events |
| Chiu | VRT + CT | CT | Nintendo Wii^®^ | Bowling, Air Sports, Frisbee, Basketball | Non-immersive | 40 min | 3 sessions per week | 6 weeks | Home | Yes | No serious adverse events |
| Daliri M(2025) | VRT + CT | CT | VR hand tracking device: Leap Motion Controller | Simulated kitchen and eating scenario | Semi-immersive | 60 min | 2 sessions per week | 6 weeks | Hospital | Yes | NA |
| El-Shamy (2018) | VRT + CT | CT | Nintendo Wii^®^ | Tennis, Boxing, Bowling, Basketball | Non-immersive | 40 min | 3 sessions per week | 12 weeks | Rehabilitation center | Yes | No serious adverse events |
| Uysal（2016） | VRT + CT | CT | Nintendo Wii^®^ | Boxing, Bowling, Basketball | Non-immersive | 30 min | 2 sessions per week | 12 weeks | Clinic | Yes | NA |
| Gözaçan Karabulut (2024) | VRT + CT | CT | Xbox Kinect | boxing,tennis,bowling,golf | Non-immersive | 45 min | 2 sessions per week | 6 weeks | Rehabilitation center | Yes | NA |
| Menekseoglu(2023) | VRT + CT | CT | Nintendo Wii^®^ | Tennis, Boxing, Basketball | Immersive | 30 min | 2 sessions per week | 12 weeks | Clinic | Yes | NA |
| Roberts(2025) | VRT + CT | CT | Hocoma ArmeoSpring Pediatric®,Tyromotion Pablo®,FitMi,Nintendo Wii®,Parrot Drones | Interactive therapy games | Non-immersive | 360min | Once daily | 10 consecutive weekdays | Care center；Pirate Camp | Yes | No serious adverse events |
| Rostami (2012) | VRT + CT | CT | VR hand tracking device: E-Link system | Interactive therapy games | Non-immersive | 90 min | 3 sessions per week | 4 weeks | Laboratory | Yes | NA |
| Saussez (2023) | VRT + CT | CT | REAtouch® | Construct buildings,fasten/unfasten buttons,or cutting modelling ingredients with knife and fork. | Semi-immersive | NA | everyday | 2 weeks | Rehabilitation center | Yes | NA |
| Şahin (2020) | VRT + CT | CT | Microsoft Kinect | Air challenge,Boxing trainer,Wall breaker,Jet run,Super kick | Non-immersive | 45 min | 2 sessions per week | 8 weeks | Clinic | Yes | NA |
| Wang (2021) | VRT + CT | CT | Nintendo Wii | Tennis, Bowling, Golf,Cooking, Tapping, Rhythmic movements | Non-immersive | 135min | 2 sessions per week | 8 weeks | Home | Yes | No serious adverse events |

VRT:Virtual Reality-based Intervention;CT:Conventional therapy
